# Plasmon-induced trap filling at grain boundaries in perovskite solar cells

**DOI:** 10.1038/s41377-021-00662-y

**Published:** 2021-10-28

**Authors:** Kai Yao, Siqi Li, Zhiliang Liu, Yiran Ying, Petr Dvořák, Linfeng Fei, Tomáš Šikola, Haitao Huang, Peter Nordlander, Alex K.-Y. Jen, Dangyuan Lei

**Affiliations:** 1 Institute of Photovoltaics/Department of Materials Science and Engineering, Nanchang University, Nanchang, 330031 China; 2 Department of Applied Physics, The Hong Kong Polytechnic University, Hung Hom, Kowloon, Hong Kong China; 3 Department of Materials Science and Engineering, City University of Hong Kong, Kowloon, Hong Kong China; 4 Institute of Physical Engineering, Brno University of Technology, Technická 2, Brno, 616 69 Czech Republic; 5 Laboratory for Nanophotonics, Department of Physics and Astronomy, Department of Electrical and Computer Engineering, Rice University, Houston, Texas 77005 USA

**Keywords:** Nanoparticles, Solar energy and photovoltaic technology

## Abstract

The deep-level traps induced by charged defects at the grain boundaries (GBs) of polycrystalline organic–inorganic halide perovskite (OIHP) films serve as major recombination centres, which limit the device performance. Herein, we incorporate specially designed poly(3-aminothiophenol)-coated gold (Au@PAT) nanoparticles into the perovskite absorber, in order to examine the influence of plasmonic resonance on carrier dynamics in perovskite solar cells. Local changes in the photophysical properties of the OIHP films reveal that plasmon excitation could fill trap sites at the GB region through photo-brightening, whereas transient absorption spectroscopy and density functional theory calculations correlate this photo-brightening of trap states with plasmon-induced interfacial processes. As a result, the device achieved the best efficiency of 22.0% with robust operational stability. Our work provides unambiguous evidence for plasmon-induced trap occupation in OIHP and reveals that plasmonic nanostructures may be one type of efficient additives to overcome the recombination losses in perovskite solar cells and thin-film solar cells in general.

## Abstract (web summary)

The incorporation of plasmonic core-shell nanostructures at the grain boundaries of perovskite films reduces the charge recombination loss through photo-brightening of trap states.

## Graphical abstract

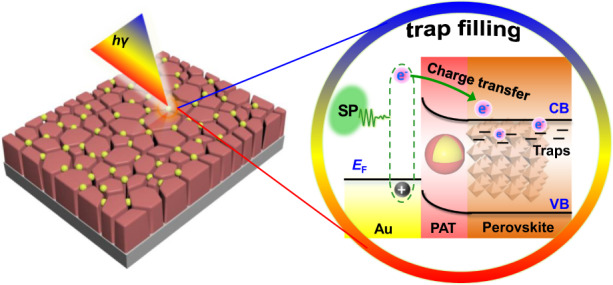

## Introduction

Undoubtedly, organic–inorganic halide perovskite (OIHP) materials have become one of the most appealing research topics in materials science and engineering^1–3^. Given the simple processability of OIHP materials, one could expect a non-negligible level of unintentional defects relevant to device operation^4^. Yet, the rate of progress in performance for perovskite solar cells (PSCs) is unprecedented, suggesting that perovskite halides have a relatively high tolerance for defect-related losses. However, intrinsic defects, such as vacancies and non-coordinated ions at their grain boundaries (GBs) and surfaces, can create deep-level traps that induce nonradiative recombination of photo-generated carriers^5^. Thus, the highest reported efficiency still falls short of the theoretical limit of ~30–33% for bandgaps in the range of 1.2–1.6 eV^6,7^. In addition, the charged defects would result in electrical instability in perovskite devices^8^. Defects thus remain one of the interesting material characteristics that underpin limitations in device operation and restrict further progress towards the theoretical limit^9,10^.

Due to their ionic nature, the charged nature of defects in OIHP materials enables unique passivation methods such as coordinate bonding or ionic bonding, to neutralize and de-activate deep-level traps^11,12^. For example, the excess electrons of under-coordinated halide ions and Pb–I antisite defects can be passivated by Lewis acids through coordinate bonding or by cations through ionic bonding, whereas the under-coordinated Pb^2+^ ions or Pb clusters can be passivated by Lewis bases through coordinate bonding or by anions through ionic bonding^13,14^. Other additives, such as polymers^15^, ionic liquids^16^, and nanomaterials^17,18^, are currently being used to alter perovskite crystal-growth dynamics and passivate defects present in perovskites. Although the incorporation of nanoparticle (NP) itself would not reduce the deep-level traps, the incorporated NPs can passivate the uncoordinated lead ions in the GBs of perovskite by their capped functional groups, such as hydroxyl and amine^17–19^.

Among all the nanomaterials, metal nanostructures showing localized surface plasmon resonances (LSPRs) with collective oscillations of free electrons in metal nanocrystals under resonant excitation^20^ have recently achieved considerable attention. Through the decay of LSPR in metallic nanostructures, the energy stored in a surface plasmon can be transferred either via a radiative path or a nonradiative path by generating hot carriers^21,22^. Previous work has demonstrated that both the plasmon-induced hot electrons and the resonant energy transfer processes can occur on a time scale of <100 fs in metal–perovskite nanocrystal complex^23^. Most studies of plasmonic PSC have focused on how the metallic nanostructures affect the photo-response of the active layer^24,25^. Yet, much less is known about the microscopic details of how the plasmon-induced interfacial processes regulates the electronic properties in the metal/perovskite heterostructures, not to mention how to take advantages of the electronic role of plasmon decay—if one exists—to improve the photoactivity of perovskite films. To ensure efficient plasmonic energy transfer^26^, the metal nanostructure must be in a region very close to perovskite or in direct contact with it, rather than mediated by charge transport materials^27^. However, direct contact would easily bring about charge recombination and exciton quenching losses at the metal surface due to dipole–dipole interactions and charge-accumulating mechanisms^28^.

On the basis of the rationale mentioned above, we modify Au NPs with ultra-thin conductive poly(3-aminothiophenol) (PAT) in situ, as *π*-conjugation phenylamine molecules have been recognized as effective adsorbing molecules with excellent charge transport properties^29^. We report that the uniform distribution of the PAT-coated gold (Au@PAT) core-shell nanostructures at the GBs of polycrystalline perovskite films can effectively reduce the charge recombination loss of PSCs under light illumination. We demonstrate that the addition of such NPs causes significant photo-induced changes in local photoluminescence (PL) and photovoltage at the GB region, corresponding to nearly an order-of-magnitude reduction in the density of deep traps. Combined experimental and theoretical investigation on the Au@PAT/perovskite heterostructures unveils that the plasmon-induced hot-electron transfer from plasmonic metal into nearby perovskite is a plausible pathway for trap filling at the perovskite GBs. The Au@PAT-containing devices enable universal efficiency enhancement and stability improvement for multiple perovskite absorbers with the best performance of 22.0% in triple-cation perovskites. Our results highlight the interplay between the rational design of metal nanostructures and plasmon decay-related charge dynamics at the metal/perovskite interface, and their collective impact on device performance.

## Results

### Preparation of core-shell plasmonic nanoparticles

**Fig. 1:**
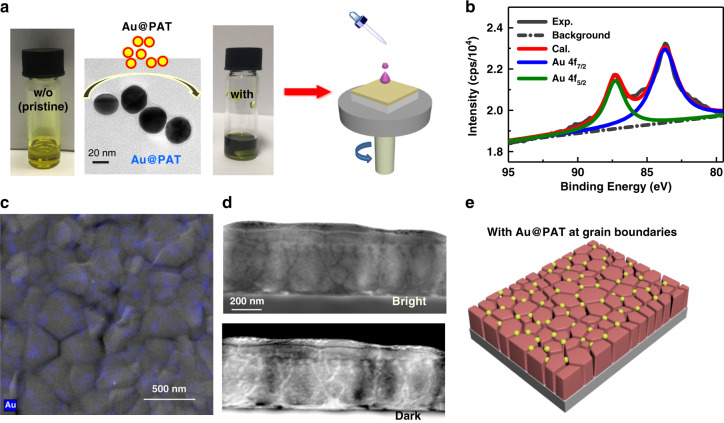
Preparation and distribution characterization of Au@PAT-modified perovskite films. **a** Addition of Au@PAT NPs (2 mg ml^−1^) into the perovskite precursor solution and the corresponding images before and after doping. The inset shows the TEM micrograph of as-synthesized Au NPs coated with ~2 nm PAT. **b** XPS spectra of Au 4*f* obtained from perovskite films with the inclusion of Au@PAT NPs (2.0 mg ml^−1^). **c** EDS mapping (in SEM mode) of Au in the Au@PAT-decorated perovskite film. **d** ABF (Bright) and HAADF (Dark) STEM images of the perovskite film with Au@PAT additives. **e** Schematic distribution of Au@PAT additives within perovskite crystals

We first had to find a suitable plasmonic nanostructure to improve the miscibility with perovskite and prevent undesired losses via charge trapping. Here we chose a specially designed coating by taking (3-aminothiophenol) as ligand to fabricate an ultra-thin and compact shell with functionalized amino groups in situ^30^. Metal core used in the present study was solution-phased Au NPs synthesized according to Turkevich procedures^31^. The transmission electron microscopy (TEM) micrograph (Supplementary Fig. S1) shows the uniformly distributed bare Au NPs with an average diameter of ~20 nm. As illustrated in Supplementary Scheme S1, 3-aminothiophenol with –SH group would be adsorbed favourably onto Au NPs due to the formation of Au–S bond. Polymerization was then proceeded by addition of oxidizing reagents to achieve PAT shell (for details, see “Methods”). To balance the need of near-field enhancement and charge-recombination suppression, we precisely controlled the core-shell nanostructures with shell thickness as thin as ~2 nm (Fig. 1a) by adjusting the molar ratio of sodium dodecylsulfate (SDS) to 3-aminothiophenol. The successful coating is confirmed by red-shifted plasmon peak from 522 to 532 nm in the absorption spectroscopy (Supplementary Fig. S2). It is worth noting that Au@PAT NPs showed good chemical stability in both acid and alkaline solutions (pH values ranging from 2 to 13). Fourier-transform infra-red spectra in Supplementary Fig. S3 and X-ray photoelectron spectroscopy (XPS) in Supplementary Fig. S4 demonstrate that amino groups are adsorbed favourably onto the as-synthesized Au@PAT NPs. It endows the Au@PAT with good dispersibility in *N*,*N*-dimethylformamide (DMF) and excellent miscibility with perovskite (Supplementary Fig. S5).

After the coating treatment, the Au@PAT NPs were intermixed with the perovskite precursor solution for film fabrication, as shown in Fig. 1a. In order to accurately and explicitly illustrate the role of plasmonic NPs, we first used methylammonium lead iodide (MAPbI_3_) perovskite material, given its simple composition and easy preparation. The successful incorporation of the metal NPs was proved through the measured Au 4*f* XPS spectrum (Fig. 1b) and the concentration of Au@PAT NPs in the MAPbI_3_ film was found close to the feeding ratio in the precursor solution (Supplementary Fig. S6). As discussed above, we speculate that the surface amino groups of Au@PAT interact with the Pb^2+^ and then act as heterogeneous nucleation sites. The results of energy dispersive X-ray spectroscopy mappings (Fig. 1c) indicate that those Au NPs (0.8 mg ml^−1^) are mainly distributed at the GBs of the modified perovskite film. In addition, the annular bright-field and high-angle annular dark-field cross-sectional scanning TEM images reveal that the perovskite crystalline grains are surrounded by metal NPs uniformly throughout the vertical direction (Fig. 1d). The nearly unchanged peak intensity of Au 4*f* in the XPS profiles before and after etching confirms the homogeneous distributions within the perovskite layer. We further used high-resolution TEM to investigate the location of the Au@PAT, which were prepared according to a transfer method without affecting the film crystalline. As shown in the TEM image (Supplementary Fig. S7) of Au@PAT-doped MAPbI_3_ films, “NP walls” mainly exist among perovskite grains. The distribution of Au@PAT additives (Fig. 1e) is consistent with the GB theory that GBs are the preferential sites for the segregation of foreigners^18^.

### Plasmonic device performance characterization

**Fig. 2:**
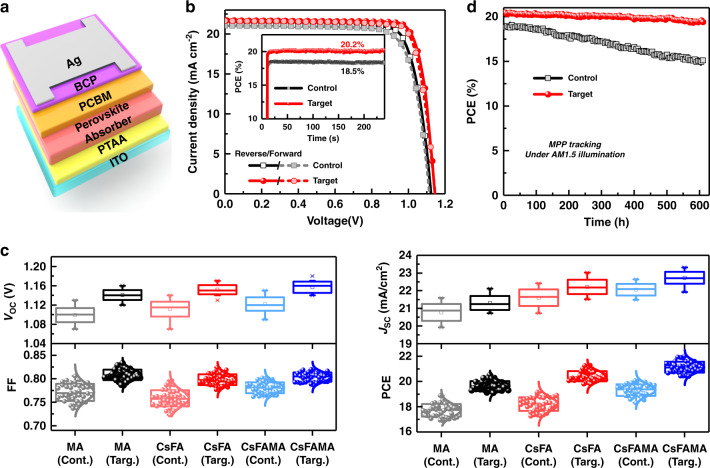
Device performance characterization. **a** Schematic of the cell architecture used in this study. **b** Current density–voltage (*J–V*) curves of the champion MAPbI_3_-based control (with PAT additives) and target (with Au@PAT additives) devices. The inset shows steady-state PCE measurement results of the best control and target MAPbI_3_ devices. **c** Statistical distributions of photovoltaic parameters for the control (Cont.) and target (Targ.) PSCs based on MAPbI_3_, CsFA, and CsFAMA perovskites (based on 40 separate measurements for each condition). **d** Long-term operational stability results of the control and target MAPbI_3_-based PSCs

Next, we investigated the influence of Au@PAT on device performance of PSCs in a planar inverted architecture, indium tin oxide (ITO)/poly(bis(4-phenyl)(2,4,6 trimethylphenyl)amine) (PTAA)/MAPbI_3_/[6, 6]-phenyl-C61-butyric acid methyl ester (PC_61_ BM)/bathocuprione (BCP)/Ag (Fig. 2a). The pristine MAPbI_3_ device exhibits a short-circuit current density (*J*_SC_) of 21.20 mA cm^−2^, an open-circuit voltage (*V*_OC_) of 1.12 V, a fill factor (FF) of 0.78 and a power conversion efficiency (PCE) of 18.59% in reverse scan (Supplementary Fig. S8). After fine-tuning the concentration of NPs from 0.4 to 2.0 mg ml^−1^ (see Supplementary Fig. S9 and Table S1), the champion device comprising 0.8 mg ml^−1^ Au@PAT presents a *J*_SC_ of 21.71 mA cm^−^^2^, a *V*_OC_ of 1.15 V, a FF of 0.82, and a PCE of 20.52%. To rule out the possibility that the enhanced performance is mainly induced by the PAT shell itself rather than the gold core, we performed a control experiment by adding PAT into the MAPbI_3_ precursor at equivalent ratios^29^. (Hereafter, the PAT-modified devices are denoted as ‘control’, whereas the Au@PAT-treated devices are denoted as “target” for convenience). Figure 2b shows the current density and voltage (*J–V*) curves of the best control and target devices. There is no significant difference between reference devices (18.59%) and control devices with PAT (18.90%). This result indicates that the gold core is essential for the observed enhancement. As shown in the statistical distribution of Fig. 2c and Supplementary Table S2, the introduction of metal NPs leads to considerable improvements in all of the photovoltaic parameters with better reproducibility. In addition, the target MAPbI_3_ PSCs achieved a stabilized PCE of 20.2% at the maximum power point (MPP), agreeing well with that obtained from the *J–V* curves. The external quantum efficiency (EQE) curves of the corresponding PSCs confirm the improved photocurrent (Supplementary Fig. S10).

To demonstrate general applicability of this method, we employed environmentally stable MA-free perovskite (Cs_0.17_FA_0.83_)Pb(I_0.8_Br_0.2_)_3_ (referred to as CsFA)^32^ and triple-cation Cs_0.05_(FA_0.83_MA_0.17_)_0.95_Pb(I_0.83_Br_0.17_)_3_ (referred to as CsFAMA)^33^ as the absorbers. For the CsFA devices, we achieved a maximum PCE of 19.23% for the control devices and a substantially increased performance of 21.28% for the target cells (Supplementary Fig. S11), whereas the best PCE of the CsFAMA-based devices was enhanced from 20.07% to 21.96% (Supplementary Fig. S12). Again, for both absorbers, all of the photovoltaic parameters are improved simultaneously (Fig. 2c) with negligible *J–V* hysteresis.

### Trap filling underpinning efficiency enhancements

We then carried out a series of optical and electrical characterizations on both control and Au@PAT-modified devices to identify major potential factors responsible for the enhanced performance. On one hand, we note that the wavelength-dependent absorption differences between the PAT-doped and Au@PAT-doped MAPbI_3_ films are consistent with their corresponding device EQE enhancements, which can thus be attributed to the LSPR effect of Au@PAT induced by light illumination. Compared with the absorption spectra of Au@PAT NPs in solvents, the film absorption enhancement peak at around 600 nm is red-shifted and broadened, due to change of refractive index (Supplementary Fig. S13) and the clustering effect of the plasmonic nanostructures at the GBs of perovskite films^34^. On the another hand, an increased PL intensity and extended PL lifetime for the Au@PAT-doped perovskite film (Supplementary Fig. S14) demonstrate a suppressed trap-state density^13,15^, which is consistent with the enhanced *V*_OC_ and FF. Determined from illumination-dependent *V*_OC_ (Supplementary Fig. S15), the reduced ideality factor of the Au@PAT-modified target cells (*n* = 1.21) compared to that of the control device (*n* = 1.41) further confirms the suppressed recombination in the plasmonic devices.

Due to effective defect passivation of perovskite films via the incorporation of Au@PAT NPs, the issues associated with perovskite degradation and ion migration^35,36^ in PSCs can be significantly alleviated, thereby improving long-term operational stability. Supplementary Fig. S16 shows X-ray diffraction (XRD) patterns of the control and target perovskite films before and after humidity aging in air under visible light. Compared with the PAT-modified film, the Au@PAT-treated sample exhibits retarded degradation with negligible feature of PbI_2_. To further investigate the operational stability, both encapsulated control and target PSCs based on MAPbI_3_ were assessed with the ISOS-L-1 protocol^37^ at ambient conditions (Fig. 2d). After 600 h continuous illumination by a 1-Sun solar simulator at MPP, the control devices rapidly lost 23% of their initial PCE, whereas the target devices exhibit a better stability by maintaining 94% of their initial PCE (slightly decreasing from 20.5% to 19.3%). Similar results of improved stability were witnessed in the CsFA perovskite devices (Supplementary Fig. S17).

We recognize that the main contributions to electronic properties of target devices may include suppression of charge recombination, facilitation of carrier extraction, and enhancement of perovskite crystallinity, etc. First, as the Au@PAT NPs are mainly distributed at the perovskite GBs, which act as a dominant source of charged defects, one may argue that the decreased trap-state density may originate from improved film quality (better crystallinity) and larger grain size (less GBs) due to the addition of Au@PAT NPs. However, we found a negligible change in perovskite morphology and crystallinity by adding Au@PAT at a low concentration as was done in this work. In particular, the pristine, control (PAT-treated), and target (0.80 mg ml^−1^ Au@PAT-modified) perovskite films all exhibit compact texturing with similar grain sizes as observed from scanning electron microscopy (SEM) micrographs (Supplementary Fig. S18). Similarly, the bulk and surface information conveyed by XRD and grazing-incidence wide-angle X-ray scattering, respectively, exhibit unaltered peak positions and intensities (Supplementary Fig. S19), indicating that neither Au clusters nor PAT perturbs the perovskite crystal lattice. We therefore rule out the film quality (crystallinity) as a dominant role for the observed performance enhancement.

Besides the degree of perovskite crystallinity, trap density and charge collection in a solar cell can also impact the device’s *V*_OC_ and FF. We quantified the trap-state densities of the reference, control, and target devices by space-charge-limited current (SCLC) measurements in the dark, which are very close to each other (2.7 × 10^16^, 2.4 × 10^16^, and 2.5 × 10^16^ cm^−3^) (Supplementary Fig. S20a). Meanwhile, we determined from time-resolved PL data^38^ that, on average, control films have a surface recombination velocity (SRV) of ∼800 cm s^−1^, whereas the Au@PAT-treated target films exhibit an average SRV of ∼700 cm s^−1^ (Supplementary Fig. S20b). In addition, we observed from ultraviolet photoelectron spectroscopy results that the energy-band alignment in perovskite layers remains unchanged after the incorporation of plasmonic NPs (Supplementary Fig. S20c). However, a notable change in the charge recombination dynamics was found from the transient photovoltage measurements (Supplementary Fig. S21). For the target devices, lifetimes span from 4 to 360 µs under a given illumination intensity, while the control devices exhibit a lifetime range from 2 to 110 µs under the same illumination condition, indicating decreased carrier recombination in the plasmonic cells under operation. This implies that the plasmon-enhanced light-molecule interaction, rather than the nanostructure itself, might be the main source of suppressed trap-state density.

### Plasmonic effects on local changes in film properties

**Fig. 3:**
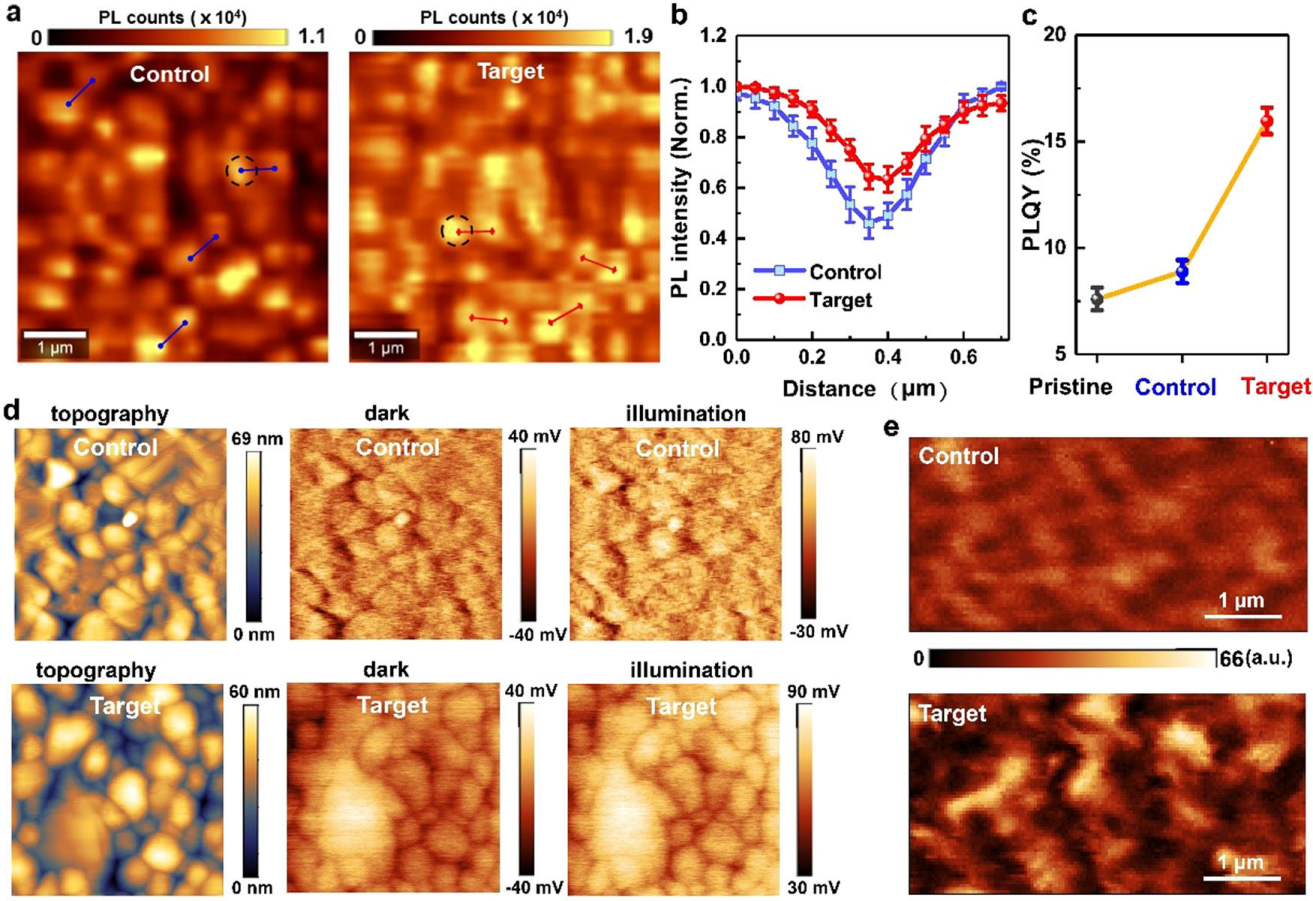
Local changes in perovskite films under illumination. **a** PL mappings of MAPbI_3_ films measured under 532 nm excitation. Control: PAT-treated MAPbI_3_. Target: Au@PAT-modified MAPbI_3_. **b** Normalized PL line scans across GBs of the control and target MAPbI_3_ films in an average of four random places (labeled in Fig. 3a). **c** PLQY of the pristine (black), control (blue), and target (red) MAPbI_3_ films in an average of five samples of each type. **d** Topography and CPD spatial maps of control and target perovskite films characterized by KPFM over an area of 4 µm^2^ under illuminated and dark states. **e** SNOM optical images showing the near-field distribution profiles of control and target perovskite films, under illumination by a 532 nm laser

As a step further, we investigated the local composition by performing nanoscale mapping to ascertain whether the plasmon-induced efficiency enhancement can be directly related to the filling of GB defect sites. Figure 3a shows confocal PL images of control and target perovskite films on quartz substrate. For the control (PAT-modified) MAPbI_3_ film, the PL intensity varies from grain-to grain, showing ~55% lower PL intensity at GBs relative to grain interiors (GIs), after deconvolution of the microscopic point-spread function (Fig. 3b). In addition, the PL spectrum collected at the dark region of a grain in the control MAPbI_3_ film is red-shifted (~4 nm) and slightly broadened compared to the bright region (Supplementary Fig. S22). Previous studies have reported a higher deep trap-state density in the dark region as compared with the bright region^5,39^. After introducing Au@PAT into the perovskite film, the entire film becomes not only brighter (~1.8-fold enhancement in PL intensity) but also more uniform with less grain-to-grain intensity fluctuation, as evidenced by <40% reduction of GB brightness relative to the GI and a decreased spectral linewidth. In addition, the PL emission shift between GI and GB is also decreased. To clearly quantify the plasmon-induced PL brightening, we determine the PL quantum yield (PLQY) of pristine, control, and target MAPbI_3_ films (Fig. 3c). As PLQY directly reflects the relative weight of radiative rate vs. the total decay rate, a significantly enhanced PLQY observed for the Au@PAT-treated sample reveals the benefit of plasmon-induced trap filling.

The photovoltaic activity of perovskite film was further studied by using Kelvin probe force microscopy, in the dark and under illumination (0.3 W m^−2^). Comparison of line profiles within this area reveals that contact potential difference (CPD) values differ between GBs and GIs, being higher under illumination due to charge accumulation. For control film, a ~140% broadening in CPD distributions (from dark to illumination) is a clear indication that the distribution of charges at the surface is broader and thus photo-induced charge generation is not uniform (Fig. 3c). Interestingly, this local difference under illumination is much reduced in the Au@PAT-modified films. The GBs “light up” indicates negligible energy barrier at the boundary and facilitated charge transport across the GBs^40,41^. Overall, those local results are direct evidences of trap filling at the GBs under illumination.

More remarkably, plasmon hybridization resulting from the near-field interaction between adjacent plasmonic nanostructures could significantly enhance the local electromagnetic field intensity at a plasmonic hotspot. We employed scattering-type scanning near-field optical microscopy (s-SNOM)^42^ to visualize the near-field distribution profile in the MAPbI_3_ film with Au@PAT NPs. The magnitude of the scattered signal probes the extent of the near-field intensity. The s-SNOM images (Fig. 3d) of the control MAPbI_3_ film under 532 nm excitation exhibited an insufficient contrast between GBs and GIs^43^, whereas the film doped with Au@PAT exhibited high scattering intensity at the GB with striking contrast. Despite the fact that it was not possible to resolve an individual metal particle due to the resolution limit (probe aperture diameter ~60 nm), we clearly observed the near-field enhancement at the GBs.

### Plasmonic effect on charge carrier behaviour

**Fig. 4:**
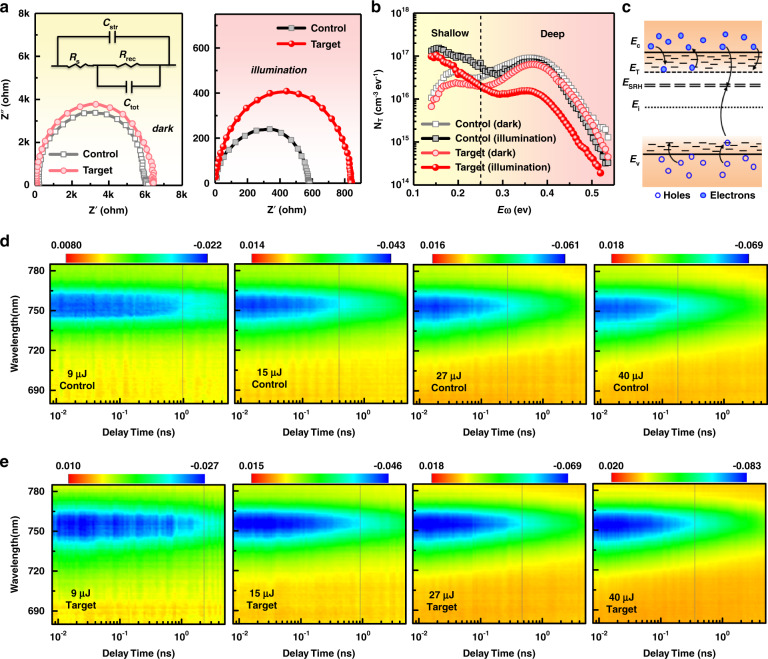
Characterization of carrier dynamics under dark and illuminated conditions. **a** Nyquist plots of EIS characterization under dark and illumination at a bias of 0.8 V for MAPbI_3_-based control and target devices. The inset shows the equivalent electrical circuit model, consisting of a single parallel connection of *R*_rec_ and a chemical capacitance (*C*_rec_) connected to a series resistance *R*_s_. **b** *t*DOS for control and target MAPbI_3_ perovskite devices in TAS analysis. **c** Energy-band diagram of the perovskite film representing the carrier recombination and trapping/detrapping processes. **d**, **e** Pseudo-colour representation of time-resolved transient absorption spectra for the control and target MAPbI_3_ films, following 532 nm pulsed laser excitation with various pump fluence

We further evaluated how the plasmon-induced local changes at the GBs impact on the carrier dynamics. A comparison of charge carrier lifetimes for the control and target MAPbI_3_ PSCs is evaluated by Nyquist plots of electrochemical impedance spectroscopy (EIS, Fig. 4a). Under illumination, the recombination resistance (*R*_rec_)^44^ of the modified devices was clearly improved as compared with the control devices. By contrast, the *R*_rec_ was just slightly increased when the devices are left in the dark. A clear trap density of state (*t*DOS) profile on the photo-response was revealed by thermal admittance spectroscopy (TAS). As shown in the Fig. 4b, the trap-state peak around 0.15 eV is generally ascribed to the shallow traps in MAPbI_3_, whereas the peak near 0.40 eV is related with deep-trap states. Under dark condition, the incorporation of Au@PAT caused a slight drop of defect density (*N*_t_) but of the same order of magnitude as for PAT-modified MAPbI_3_, consistent with SCLC results. Under illumination, we observed a reasonable change of *t*DOS in TAS analysis for the control device^45^. Surprisingly, the device with Au@PAT NPs exhibited a significantly lower integrated trap-state density of 0.5 × 10^16^ cm^−3^ across the whole deep-level, as compared with 2.1 × 10^16^ cm^−3^ of the control cell.

As shown in Fig. 4c, the trap states stemming from deep levels, rather than shallow levels, serve as major recombination centres (*E*_SRH_), as the trapped charges there are more likely to be annihilated or recombined with an opposite carrier. In general, the trap-assisted Shockley–Read–Hall (SRH) recombination rate *R*_SRH_ can be expressed as^46^

1 $$R_{{{{\mathrm{SRH}}}}} = V_{{{{\mathrm{th}}}}}\sigma {\rm N}_{{{\mathrm{t}}}}\frac{{np - n_{{{\mathrm{i}}}}^2}}{{n + p + 2n_{{{\mathrm{i}}}}{{{\mathrm{cosh}}}}\left( {\frac{{E_{{{\mathrm{t}}}} - E_{{{\mathrm{i}}}}}}{{k_{{{\mathrm{B}}}}T}}} \right)}}$$

here, *σ* is the capture cross-section of the traps, *n*_i_ is the intrinsic carrier concentration, *E*_t_ is the trap energy level, *E*_i_ is the energy of Fermi level, *n* is the electron concentration, *p* is the hole concentration, *k*_B_ is the Boltzmann constant, and *T* is the absolute temperature. Thus, the deep-level trap occupation induced by Au@PAT may lead to a longer charge carrier lifetime and lower SRH recombination rate in the device that enhances the FF and *V*_OC_.

To elucidate the defect-mediated recombination dynamics, we further measured transient absorption (TA) spectra from the picosecond to nanosecond regime. Figures 4e, f show the pseudo-colour TA maps of MAPbI_3_ films with PAT (control) and with Au@PAT (target) as a function of excitation density from 9 to 40 μJ cm^−2^, respectively. The negative absorption change centred on 755 nm is attributed to the photobleaching that arises from the band filling effect^47^. It is evident from all the TA plots that the decay of the bleach signal shows a strong dependence on the plasmonic additives, especially for the excitation with low pump fluence; the bleaching persists to longer times for the target perovskite film than that for the control film. Moreover, the recombination dynamics can be described by the following rate equation^48^:

2 $$\frac{{\partial n}}{{\partial t}} = G - k_1n - k_2n^2 - k_3n^3$$

where *G* is the charge-density generation rate, *k*_1_ is the monomolecular recombination rate, *k*_2_ the bimolecular recombination rate, and *k*_3_ the Auger recombination rate constant. Here, *k*_1_ was extracted by a single exponential fit of the tail of time-resolved PL data at the time scale of 100 ns^49^. The *k*_2_ and *k*_3_ were extracted by carrying out global fits of our data at varied fluence (see Supplementary Fig. S23). For the control MAPbI_3_ film, the first-, second-, and third-order recombination rate constants are determined as 2.3 ± 0.7 × 10^7^ s^−1^, 3.2 ± 0.4 × 10^−10^ cm^3^ s^−1^, 1.7 ± 0.2 × 10^−28^ cm^6^ s^−1^, respectively, whereas for the target film, the *k*_1_, *k*_2_, and *k*_3_ values are 1.5 ± 0.6 × 10^7^ s^−1^, 1.2 ± 0.5 × 10^−10^ cm^3^ s^−1^, 2.1 ± 0.2 × 10^−28^ cm^6^ s^−1^, respectively. The significantly lower rate constants (*k*_1_ and *k*_2_) for the modified perovskite clearly confirm that the presence of plasmonic NPs can lower charge recombination rates and enlarge carrier diffusion lengths.

Besides charge kinetics, carrier thermalization may also be influenced by the presence of Au@PAT, as the decrease in defect density suppresses relaxation pathways for the hot carriers^50,51^. Notably, our target MAPbI_3_ films exhibited slightly longer hot-carrier cooling lifetimes (*T*_c_) than those of the control films under similar *n*_0_ (see Supplementary Figs. S24 and S25). In addition, the sub-bandgap TA signal^52^ at 1.58 eV also shows slower decay after Au@PAT incorporation (Supplementary Table S3). It convinces us that the addition of Au@PAT in the perovskite film enables effective tailoring of charge carrier dynamics in PSCs.

## Discussion

These significant contrasts of charge carrier behaviour between the control and target devices under dark and illumination confirm that the related deep-trap filling is plasmon-induced rather than the nanostructure itself. The origin behind the effect is very different from many traditional passivation methods such as coordinate or ionic bonding, and we then turned to understand how the plasmon-induced effect could efficiently suppress the charged defects. Two distinct plasmonic mechanisms may play a role in modulating electronic properties of metal/perovskite interfaces^53^. In plasmon-induced resonance energy transfer (PIRET)^54^, the absorbed energy in a plasmonic metal can be transferred into an adjacent semiconductor through an excitation of an electron (Supplementary Scheme S2a). Another possibility is plasmon decay into hot carriers, which can then be transferred into the adjacent semiconductor, a process referred to as plasmon-induced hot-electron transfer (PHET) pathway (Supplementary Scheme S2b)^26,55^.

**Fig. 5:**
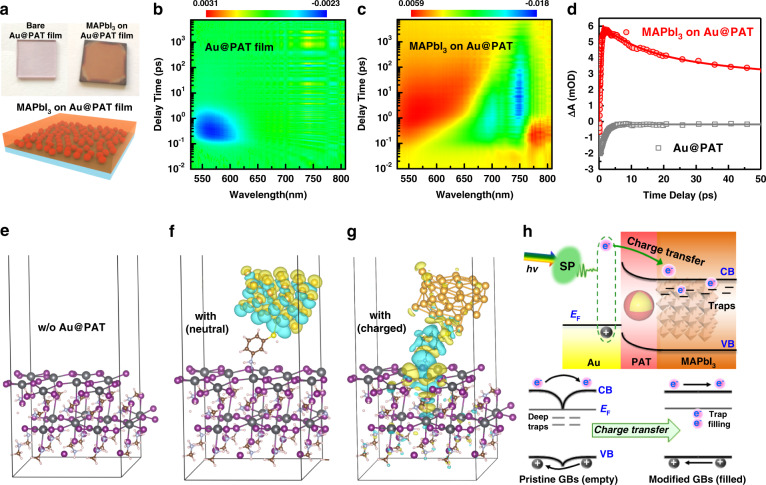
Proposed mechanism for photo-induced plasmonic cleaning. **a** The optical images of the Au@PAT film assembled from colloidal solution and the Au@PAT/MAPbI_3_ bilayer film. The schematic below shows the bilayer structure of perovskite deposited on Au@PAT film assembled on quartz glass. **b**, **c** Pseudo-colour TA spectra of Au@PAT films on bare glass substrates without and with MAPbI_3_ film deposition under photoexcitation wavelength of 532 nm. **d** Charge dynamics probed at the 580 nm (bleach peak of Au@PAT film) of bare Au@PAT and Au@PAT/MAPbI_3_ bilayer samples. **e**–**g** Theoretical calculations of intermolecular interactions using density functional theory (DFT). Charge-density difference distribution of the (001) slab of untreated MAPbI_3_ surface with under-coordinated Pb defects (**e**), 3-aminothiophenol-capped Au_38_ cluster on MAPbI_3_ surface without additional charge (**f**), and charged by one additional electron (**g**). Charge accumulation and depletion are shown in yellow and blue, respectively. Au, Pb, I, C, N, S, and H are denoted in golden, grey, purple, brown, cyan, yellow, and pink spheres, respectively. **h** Schematic depicting the PHET mechanism at Au@PAT/MAPbI_3_ interfaces for trap filling. The carrier-dynamic diagrams at the GBs before and after the hot-electron injection from Au@PAT is presented

Hot-electron injection by charge transfer across ultra-thin conductive PAT layer into the surface of perovskite grains is possible because of their strong metal/semiconductor binding and suitable band alignment^56^. To decide whether hot-electron transfer exists, we maximized hot-carrier production by preparing a self-assembled NP film (Supplementary Fig. S26) for a bilayer heterostructure (Fig. 5a). The TA spectra for the bare Au@PAT film (Fig. 5b) exhibit an exponential signal characteristic of hot-carrier thermalization near a resonance, which is frequently observed in metallic structures. For the Au@PAT/MAPbI_3_ bilayer film (Fig. 5c), we observed much broader photon-induced absorption (PIA) signals with no trace of the localized thermalization for the Au@PAT film apparent in Fig. 5b. The PIA features show crucial differences compared with MAPbI_3_ thin layer on bare fluorine-doped tin oxide (FTO) (Supplementary Fig. S27). With an efficient carrier injection process (<1 ps) and long lifetimes in the bilayer hybrid films (Fig. 5d and Table S4), the changes in PIA corresponds to a change in the hot-carrier population in the MAPbI_3_ acceptor^26^. Thus, we attribute the positive PIA signal in Au@PAT/MAPbI_3_ bilayer to hot carriers that have been transferred from the plasmonic particles to the CB of MAPbI_3_. The PIA signal is wavelength dependent^23^ and a less efficient plasmon-induced hot-carrier transfer is identified for an excitation wavelength of 365 nm, far from the LSPR absorption region (Supplementary Fig. S28). These findings further support the importance of the LSPR for enhancing the electronic properties of nearby perovskites.

First-principles density functional theory calculation was employed to gain further insight into the electronic structure of the defect annihilation under the PHET process. Here we proposed a simplified model consisting 3-aminothiophenol-capped Au cluster (Au_38_)^57^ on top MAPbI_3_ surface with partial loss of MAI^13^. For bare MAPbI_3_, the electron distribution was localized around the deep defect states of Pb_I_ site on the surface (Fig. 5e). When 3-aminothiophenol-capped Au_38_ cluster was absorbed on the surface of perovskite, we found negligible charge transfer due to chemisorption (Fig. 5f). This finding further confirms that the incorporation of functionalized NPs alone cannot passivate the deep-trap states effectively. However, when Au_38_ cluster was charged with one additional electron to simulate the generation of plasmon-induced hot carriers^58^, the electron distribution of perovskite surface becomes much more delocalized with an increased overlap of wave functions (Fig. 5g). The Bader charge analysis shows that the Au clusters can transfer about 0.97 electrons to the under-coordinated Pb defects. Thus, the hot electrons in plasmonic Au nanostructures could inject into trap sites across the PAT shell, making it a better Lewis base relative to the amine group^29^. Therefore, we assumed that the deep traps at GB when filled with hot electrons are likely to be electrically neutral^59^. The trap filling by PHET process effectively reduces the trap-assisted recombination in the device, as shown in Fig. 5h. In addition, the charge transfer would become more obvious when we take local electric field enhancement induced by LSPR into consideration^58^.

The observation of charge transfer from metal to semiconductor within the heterostructure is a robust feature of the PHET process. Due to the presence of strong plasmon–exciton coupling, PIRET may also play a role^23,56^. To investigate that, we compare PL excitation (PLE) spectra for bare MAPbI_3_ and Au@PAT/MAPbI_3_ bilayer films (Supplementary Fig. S29). The PLE spectrum showed only a slight increase of the PL signal after the incorporation of Au@PAT. If PIRET process would have made a significant contribution to the energy transfer, we would have expected to see a significant enhancement in the perovskite emission intensity, because the perovskite excitons induced by PIRET will recombine through the same pathway. These results reveal that PHET process is a more plausible mechanism in the present system. Although this primary investigation is not exhaustive in terms of all possible contribution and direct observation of deep-trap filling in PSCs, it does highlight the effect of plasmonic resonances on metal/perovskite heterostructure.

In summary, this work clearly shows that the deep traps of PSCs can be effectively filled by positioning plasmonic Au@PAT NPs into the perovskite absorber. The key to this core-shell nanostructure design is achieving direct plasmonic coupling between metal and perovskite GBs across the ultra-thin conductive PAT polymer. Under illumination, the relatively positive local changes in photophysics are particularly significant for the GB regions on the microscopic level, playing a critical role in the reduction of deep-trap densities. The origin of the plasmonic effect appears to be an efficient plasmon-induced hot-electron transfer process. We achieved a considerable performance improvement in a plasmonic solar cell with significant *V*_OC_ and FF gains, resulting in a best PCE of 22.0%. Overall, these observations suggest that plasmonic energy conversion at the metal/perovskite heterointerfaces can be efficiently regulated and generally utilized. We foresee that further clarifications of plasmon resonance effects will allow PSCs to reach their thermodynamic limit.

## Materials and methods

### Materials

The ~20 nm Au NPs were prepared through a citrate reduction approach. Specifically, 2 ml HAuCl_4_ (10 mM) was mixed with 78 ml deionized water water and 8 ml trisodium citrate (40 mM). The solution was heated until boiling under vigorously stirring. Then the citrate-stabilized gold NPs were functioned with the amino group using the PAT shells^30^. The gold NPs were centrifuged and concentrated to 1 mM in SDS solution (20 ml, 12.85 mM), followed by adding 400 μl 3-aminothiophnol in ethanol (1 : 80). The solution was stirred for over 1 h, then 0.5 ml (NH_4_)_2_S_2_O_8_ solution (0.125 M) was added at 70 °C. The reaction mixture was incubated at 70 °C for 2.5 h to ensure complete polymerization.

### Perovskite precursor preparation

Taking MAPbI_3_ system for example, a small amount of PAT-capped Au NPs were mixed with the perovskite precursor (MAI and PbI_2_) for device fabrications in DMF : DMSO (4 : 1). A volume of 200 μl of each DMF solution of Au@PAT concentrations of 1, 2, 4, 6, or 10 mg ml^−1^ was mixed with 800 μl of the DMF : DMSO (3 : 1) solution of perovskite precursor (molar ratio of MAI and PbI_2_ is 1 : 1), for 0.2, 0.4, 0.8, 1.2, or 2.0 mg ml^−1^ of Au@PAT, respectively.

### Device preparation

The patterned ITO-coated glass (~1.5 cm × 1.5 cm) was cleaned by sequential sonication in soap solution, water, acetone and isopropanol for 20 min each and then dried under N_2_ flow and treated by oxygen plasma for 15 min. To prepare the *p*−*i*−*n* planar structure, PTAA film (8 nm) with a concentration of 2 mg ml^−1^ dissolved in toluene was spin-coated at the speed of 6000 r.p.m. for 35 s. The substrate was then post-annealed at 100 °C for 10 min. Considering the poor wetting ability of the PTAA surface, DMF solvent or an interfacial compatibilizer poly[9,9-bis((*N*,*N*,*N*-triethylammonium)-hexyl)-2,7-fluorene] dibromide was first spun onto the PTAA layer to improve the wetting ability before depositing the perovskite layer. The MAPbI_3_ was prepared dissolving PbI_2_/MAI (1 : 1 molar ratio) in DMF : DMSO (3 : 1). One hundred millilitres of the solution was spin-coated at 4000 r.p.m. for 40 s and 300 µl of diethyl ether was casted for 8 s after initiating spin-coating. The CsFA precursor was prepared by dissolving FAI, CsI, PbI_2_, and PbBr_2_ in DMF : DMSO (3 : 1) to obtain a stoichiometric solution^32^. One hundred microlitres of the precursor was dripped onto the PTAA substrates at a speed of 4000 r.p.m. (with a ramping rate of 2000 r.p.m. s^−1^) for 40 s. Three hundred microlitres of diethyl ether as the antisolvent was dripped onto the perovskite 10 s after starting the spin-coating programme. Triple-cation CsFAMA perovskite film was formed according to our previously reported procedure by mixing CsI, FAI, MABr, PbI_2_, and PbBr_2_ in DMF/DMSO (*v*/*v* = 4 : 1) to a chemical formula^33^. Precursor solution was spread onto substrates and spin-coated at 1000 r.p.m. for 10 s followed immediately by 4000 r.p.m. for 35 s. After 10 s of the second stage, 120 μL of chlorobenzene was poured on top of the substrates quickly during spin-coating. All the perovskite films were then immediately transferred onto a hotplate and heated at 100 °C for 30 min. After annealing, a solution of PC_61_BM in chlorobenzene (25 mg ml^−1^) was spin-coated on top of the perovskite film at a rotation speed of 1500 r.p.m. for 40 s. Finally, the samples were transferred to an evaporation chamber, and BCP (8 nm) and Ag (120 nm) were deposited under vacuum.

### Characterization

Optical absorption measurements were carried out in a Lambda 750 UV/Vis spectrophotometer. PL was measured using a Horiba Fluorolog time-correlated single-photon counting system with photomultiplier tube detectors. The light was illuminated from the perovskite film side. The excitation source is a laser diode with a wavelength and frequency of 480 nm and 1 MHz, respectively. Optical images of laser confocal microscopy were conducted on Olympus LEXT OLS4100. The impedance spectrum was measured using an electrochemical workstation (EC-Lab, SP300) at different frequencies, ranging from 1 MHz to 100 Hz with 100 data points. Capacitance−voltage (*C*–*V*) measurement was performed using an Autolab potentiostat (model PGSTAT30) equipped with a frequency analyzer module. For the *C*–*V* measurement, a small voltage perturbation (20 mV r.m.s.) was applied at 10 kHz. The fs-TA measurements were carried out on a Helios pump–probe system (Ultrafast Systems LLC) coupled with an amplified femtosecond laser system (Coherent, 35 fs, 1 kHz, 800 nm). The probe pulses (from 560 to 850 nm) were generated by focusing a small portion of the fundamental 800 nm laser pulses into a 1 mm CaF_2_. The power of the pump beam was modulated using a variable neutral density filter and monitored using a Thor Labs PM100 power meter. The 365 and 532 nm pump pulses were generated from an optical parametric amplifier (TOPAS-800-fs). The perovskite samples were placed in an N_2_-filled chamber. s-SNOM (NtegraSolaris NT-MDT) has been carried out in the collection mode to visualize the near-field change. The sample-tip distance was held constant (∼10 nm) by a feedback mechanism, similarly to the common non-contact atomic force microscopy mode. The excitation laser beam (wavelength 532 nm, output power 15 mW) was guided by a multimode optical fibre into an inverted optical microscope and focused on a sample structure by an objective lens. High-resolution SEM images were obtained using the JSM 6701F field-emission SEM with an accelerating voltage of 1 kV. The TEM measurements were performed on JEOL-2100F transmission electron microscope.

The devices were measured immediately after metal deposition without any preconditioning. *J*–*V* characteristics were recorded in ambient environment using a Keithley 2400 source meter under the illumination of the solar simulator (SS-F5-3A, Enli Tech) at the light intensity of 100 mW cm^−2^, with 0.1 V s^−1^ sweeping rates and a 10 mV voltage step. The power output of the lamp was calibrated using a certified silicon diode. The *J*–*V* curves were measured by forward (−0.2 V to 1.3 V) or reverse (1.3 V to −0.2 V) scans. The stabilized power output was measured by measuring the current, while holding the voltage at the MPP, which was determined from the *J*–*V* curve in the reverse scan. All devices in this study have an unmasked active area of ~0.185 cm^2^. The final active area is 0.108 cm^2^ defined by the shadow mask. The devices were measured in both nitrogen atmosphere and ambient air (humidity of 30–60%; at Nanchang), and no obvious difference was observed. The EQE spectra were obtained from a specially designed system (Enli Tech) setup consisting of a Xenon lamp (Oriel, 450 W) as the light source, a monochromator, a chopper with a frequency of 100 Hz, a lock-in amplifier, and a Si-based diode for calibration. The integrated *J*_SC_ was carefully checked and matched well with the *J*_SC_ from the *J*–*V* sweep.

## Supplementary information

Supplementary Information
