# Author Correction: Plasmon-induced trap filling at grain boundaries in perovskite solar cells

**DOI:** 10.1038/s41377-022-00712-z

**Published:** 2022-01-18

**Authors:** Kai Yao, Siqi Li, Zhiliang Liu, Yiran Ying, Petr Dvořák, Linfeng Fei, Tomáš Šikola, Haitao Huang, Peter Nordlander, Alex K.-Y. Jen, Dangyuan Lei

**Affiliations:** 1 Institute of Photovoltaics/Department of Materials Science and Engineering, Nanchang University, Nanchang, 330031 China; 2 Department of Applied Physics, The Hong Kong Polytechnic University, Hung Hom, Kowloon, Hong Kong China; 3 Department of Materials Science and Engineering, City University of Hong Kong, Kowloon, Hong Kong China; 4 Institute of Physical Engineering, Brno University of Technology, Technická 2, Brno, 616 69 Czech Republic; 5 Laboratory for Nanophotonics, Department of Physics and Astronomy, Department of Electrical and Computer Engineering, Rice University, Houston, Texas 77005 USA

**Keywords:** Nanoparticles, Solar energy and photovoltaic technology

Correction to: *Light: Science & Applications*

10.1038/s41377-021-00662-y, published online 28 October 2021

In the version of this article initially published, there were errors in Fig. 3a. The PL mapping image of Target sample (Au@PAT-modified MAPbI_3_) after treatment was incorrectly positioned on the Control sample (PAT-treated MAPbI_3_). In addition, the color scales were drawn in the wrong orientation.

So we would like to correct Fig. 3 in the initial published version to the following figure:

**Figure:**
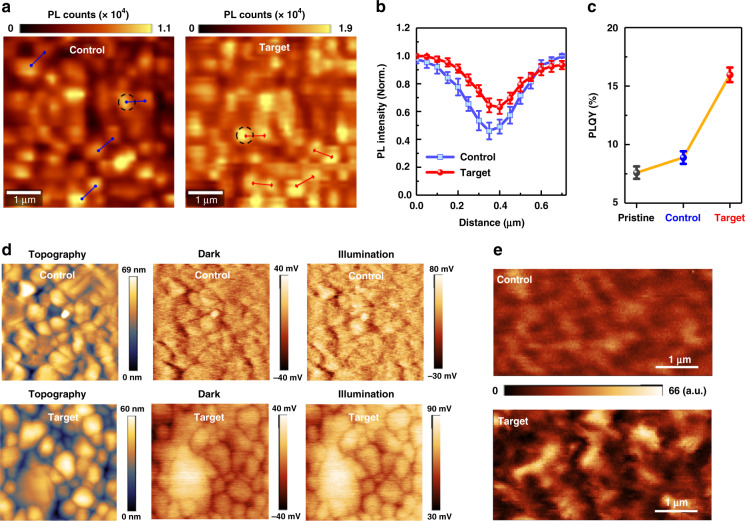

We would like to apologize for any inconvenience this may have caused.

The original article has been updated.

