# Supplementary material for: Plasmon-induced trap filling at grain boundaries in perovskite solar cells
Source: Light Sci Appl. 2021 Oct 28;10:219. doi: 10.1038/s41377-021-00662-y (PMC8553803; doi:10.1038/s41377-021-00662-y)
Supplement: Supplementary file 1 — Supplementary Information [file 41377_2021_662_MOESM1_ESM.docx]

Supplementary Information for

**Plasmon-Induced Trap Filling at Grain Boundaries in Perovskite Solar Cells**

Kai Yao^1,2^*, Siqi Li^2,3^, Zhiliang Liu^1^, Yiran Ying^2^, Petr Dvořák^2,4^, Linfeng Fei^1,2^, Tomáš Šikola^4^, Haitao Huang^2^*, Peter Nordlander^5^, Alex K.-Y. Jen^3^ and Dangyuan Lei^3^*

Correspondence: Kai Yao (yaokai@ncu.edu.cn) or Haitao Huang (aphhuang@polyu.edu.hk) or Dangyuan Lei (dangylei@cityu.edu.hk)

^1^Institute of Photovoltaics/Department of Materials Science and Engineering, Nanchang University, Nanchang 330031, China

^2^Department of Applied Physics, The Hong Kong Polytechnic University, Hung Hom, Kowloon, Hong Kong, China ^3^Department of Materials Science & Engineering, City University of Hong Kong, Kowloon, Hong Kong, China ^4^Institute of Physical Engineering, Brno University of Technology, Technická 2, Brno 616 69, Czech Republic ^5^Department of Physics and Astronomy, Department of Electrical and Computer Engineering, and Laboratory for Nanophotonics, Rice University, Houston, Texas 77005, USA

These authors contributed equally: Kai Yao, Siqi Li and Zhiliang Liu.

**1. *SCLC measurement***: The dark *J–V* characteristics of the electron-only devices were measured by a Keithley 2400 source and the trap state density was determined by the trap-filled limit voltage. The dependence of current on the applied voltage shows three regions: the ohmic region, trap filled limit (TFL) region, and child region. In the TFL region, the density of trap states (*N*_T_) can be evaluated by,

$$N_{T}={{\varepsilon\varepsilon}_{0}V_{\mathrm{TFL}}}/{eL^{2}} (S1)$$

where *ε* and *ε*_0_ are the dielectric constants of perovskite and vacuum permittivity, respectively, *e* is the elementary charge, and *L* is the thickness of the perovskite film.

**2.** ***TAS measurement***: Trap density (*N*_T_) of control and target MAPbI_3_ films is estimated from angular frequency dependent capacitance using^1-3^

$$N_{T}\left( E_{\omega} \right)=-\frac{V_{bi}}{qw}\frac{dC}{d\omega}\frac{\omega}{kT} (S2)$$

where *V*_bi_ denotes the built-in potential, *W* is the depletion width, *C* is the capacitance, *ω* is the frequency, *k* is Boltzmann constant, and *T* is the temperature. *V*_bi_ and *ω* are obtained from
*C*^-2^–*V* plot as described elsewhere. The applied angular frequency *ω* defines the energy demarcation,

$$E_{\omega}=k_{B}T\ln(\frac{\omega_{0}}{\omega}) (S3)$$

Assuming that the distribution of defect states is within a small range, the defect activation energy *E*_ω_ deduced from the Arrhenius plot is approximately the energy difference between the valence band edge (*E*_V_) and the trap state (*E*_T_).

**3.** ***Other Characterization***: GIWAXS measurements were carried out at beamline 7.3.3 of the Advanced Light Source, Lawrence Berkeley National Laboratory. Samples were measured at a detector distance of 0.249 m using X-ray wavelength of 1.240 Å, at 0.24° incident angle with respect to the substrate plane. Scattering intensity was detected by a PILATUS 2M detector. Nika software package was used to get the 2D GIWAXS images. PLQE of MAPbI_3_ films was
performed with a 532 CW laser. The integrated PL intensity was taken from 1.91 to 1.46 eV, measured in a Labsphere integrating sphere. Transient photovoltage measurements were performed at steady state under continuous illumination. A white-light halogen lamp was employed to vary the charge density. A small perturbation was induced with a 532 nm pulsed laser (pulse duration shorter than 4 ns) to keep a small voltage perturbation. After the pulse, the voltage decays back to its steady state value in a single exponential decay. The light soaking stability was tested on a home-made illumination resistance test system equipped with a Class AAA solar simulator and a Keithley for automatic recording of the *J–V* curves. The devices without encapsulation were stored in a cabinet onto a hot plate in ambient conditions. The light-soaking stability test was measured at continuous illumination under AM 1.5G.

**4. *Computational details*:** First-principles density functional theory (DFT) calculations were conducted by applying the projector augmented wave method implemented in the Vienna ab initio simulation Package^4,5^. The generalized gradient approximation with Perdew-Burke-Ernzerhof parametrization^6^ was used as the exchange-correlation functional. Kinetic energy cut-off was set as 400 eV and only Gamma point was used during the geometric optimization, while a 3 × 3 × 1 mesh with the Monkhorst-Pack sampling was applied during the self-consistent field calculations. The convergence criteria for energy and force were 1 × 10^-4^ eV and 0.05 eV Å^-1^, respectively. The slab model was constructed by the PbI_2_-terminated (001) surface with tetragonal MAPbI_3_ in a 2 × 2 supercell. Vacuum layer with the thickness of 20 Å was added to avoid the spurious interaction between adjacent image cells. Bader charge analysis^7^ was used to analyze the charge transfer process.

**Fig. S1.** TEM image of bare Au nanoparticles.

**Scheme S1.** The proposed procedure for the synthesis of Au nanoparticle coating with PAT-shell. During polymerization, the shell was functionalized with amino groups in situ.

Note: Prior to adding plasmonic nanoparticles in the perovskite precursor solution, several critical problems must be overcome. The surfaces of the bare Au nanoparticles were capped with sodium citrate, which limits the solubility of Au nanoparticles in the polar solvents for perovskite precursors, such as dimethylformamide (DMF) and dimethyl sulfoxide (DMSO).

**Fig. S2.** Absorption spectra of 20 nm Au with and without shell coating in various solvents.

**Fig. S3.** Fourier transform infrared spectra of Au@PAT NPs.

**Fig. S4.** The N 1s XPS spectrum of Au@PAT NPs. The peaks located at 398.9 and 400.2 eV are assigned to the amino group of 3-aminothiophenol and poly(3-aminothiophenol) attached onto the Au spheres, respectively.

**Fig. S5.** Optical images of pure Au@PAT NPs solution in DMF solution (0.2 and 5.0 mg ml^-1^) and MAPbI_3_ perovskite precursor (DMF:DMSO = 4:1) with the incorporation of Au@PAT NPs (2 mg ml^-1^) before and after aging for 12 hours.

**Fig. S6.** XPS survey of Au@PAT-treated (2.0 mg ml^-1^) MAPbI_3_ films (left). XPS spectra of the Au 4f and Pb 4f regions for the same set of modified sample (right). The concentration of Au in the perovskite film can be calculated through the equation

$$\frac{n_{i}}{n_{j}}=\frac{A_{i}/S_{i}}{A_{j}/S_{j}}$$

where the *A* is the integrated area and *S* is the sensitivity factor of the element (5.24 and 6.968 for Au and Pb, respectively). Thus, we can achieve the ratio of Au/Pb = 0.0115.


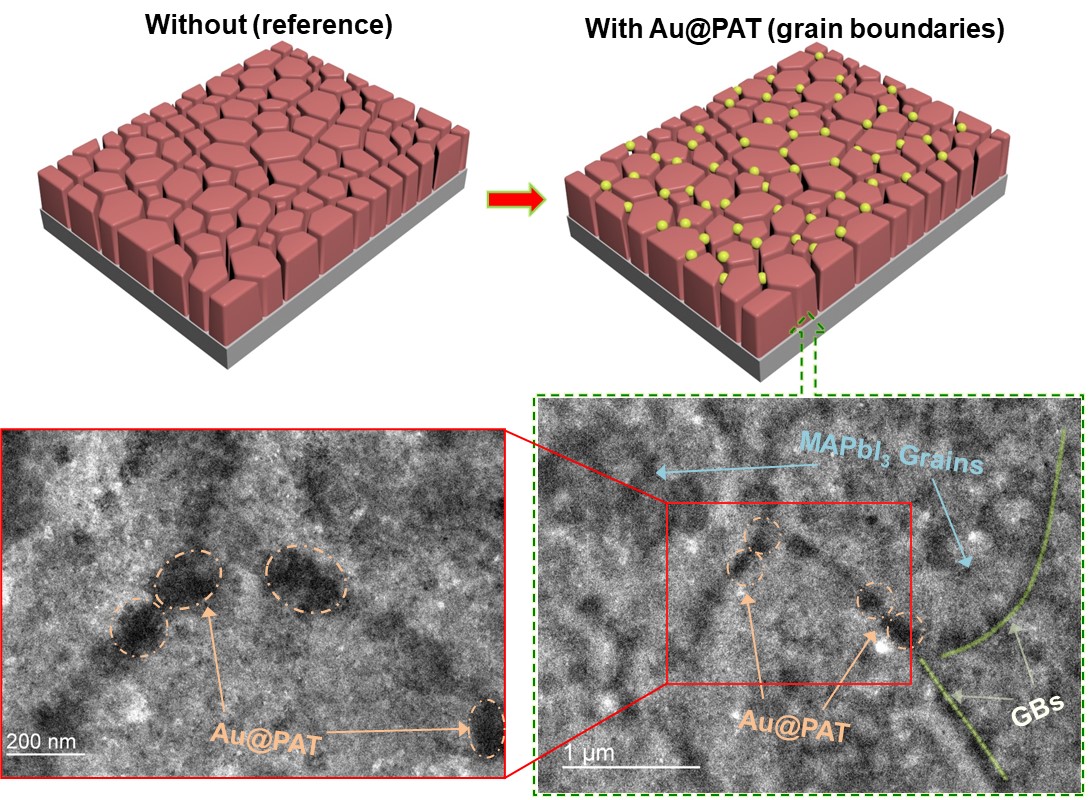


**Fig. S7.** Schematics of distribution of Au@PAT additives within perovskite film. The distribution of NPs at GBs is supported by TEM images of the thin MAPbI_3_ film (0.2 M) with 2.0 mg ml^-1^ Au@PAT NPs.

Note: To prepare the TEM sample, a poly(methyl methacrylate) (PMMA) precursor solution (100 mg ml^-1^ in chlorobenzene) was spin-coated on indium tin oxide (ITO) to form the PMMA layer, followed by the depositions of PEDOT:PSS and perovskite films. The PMMA served as a sacrificing layer and was removed by soaking in chlorobenzene, which is a non-solvent to PEDOT:PSS and perovskite, leaving a floating film of PEDOT:PSS/perovskite that was then picked up by a TEM grid for the study.^[8]^

**Fig. S8.** The *J-V* curves of pristine MAPbI_3_ device without any additives.

**Fig. S9.** Optical images of MAPbI_3_ perovskite precursor (DMF:DMSO = 4:1) with incorporation of Au@PAT NPs of different concentrations ranging from 0 to 1.2 mg ml^-1^. The image of concentration up to 2.0 mg ml^-1^ was shown in Fig. S5. (b) *J*–*V* curves measured by reverse scan for solar cells fabricated as a function of added Au@PAT contents in MAPbI_3_. Here “w/o” stands for “without”. (c) The dependence of PCE values on the PAT or Au@PAT nanoparticle concentrations as extracted from the *J-V* curves. Even under optimized concentration of PAT (0.4 mg ml^-1^), the control device showed slightly increased FF. However, the *V*_OC_ and *J*_SC_ remained almost unchanged.

**Table S1.** Performance of champion MAPbI_3_ PSCs as a function of Au@PAT concentration under AM 1.5 G illumination with a device area of 0.108 cm^2^.

| Concentration  (mg ml^-1^) | *J*_SC_  (mA cm^−2^) | *V*_OC_ (V) | FF (%) | PCE (%) | Average  PCE (%)^a^ |
| --- | --- | --- | --- | --- | --- |
| 0 (pristine) | 21.20 | 1.12 | 78.29 | 18.59 | 17.66 ± 0.53 |
| 0.4 | 21.43 | 1.14 | 80.88 | 19.76 | 18.89 ± 0.41 |
| 0.8 | 21.71 | 1.15 | 82.17 | 20.52 | 19.72 ± 0.37 |
| 1.2 | 21.18 | 1.14 | 81.18 | 19.58 | 18.74 ± 0.44 |
| 2.0 | 20.45 | 1.10 | 76.92 | 17.30 | 16.29 ± 0.61 |

^a)^The average device performance with standard deviation were obtained on the basis of 40 cells.

**Table S2.** Photovoltaic parameters of control and target PSCs measured under standard AM 1.5 G illumination with a device area of 0.108 cm^2^.

| Conditions | Sweep | *J*_SC_  (mA cm^−2^) | *V*_OC_ (V) | FF (%) | PCE  (%) | Average  PCE (%)^a^ | hysteresis index (HI)^b^ |
| --- | --- | --- | --- | --- | --- | --- | --- |
| MAPbI_3_  (Control) | Reverse | 21.25 | 1.12 | 79.42 | 18.90 | 17.96 ± 0.43 | 0.030 |
|  | Forward | 21.03 | 1.11 | 77.79 | 18.16 | 17.31 ± 0.45 |  |
| MAPbI_3_  (Target) | Reverse | 21.71 | 1.15 | 82.17 | 20.52 | 19.72 ± 0.37 | 0.018 |
|  | Forward | 21.64 | 1.14 | 81.31 | 20.06 | 19.27 ± 0.39 |  |
| CsFA  (Control) | Reverse | 21.91 | 1.13 | 77.68 | 19.23 | 18.22 ± 0.51 | 0.032 |
|  | Forward | 21.87 | 1.12 | 75.64 | 18.53 | 17.37 ± 0.49 |  |
| CsFA  (Target) | Reverse | 22.61 | 1.16 | 81.13 | 21.28 | 20.47 ± 0.40 | 0.015 |
|  | Forward | 22.53 | 1.16 | 80.21 | 20.96 | 20.09 ± 0.41 |  |
| CsFAMA (Control) | Reverse | 22.24 | 1.13 | 79.87 | 20.07 | 19.03 ± 0.46 | 0.035 |
|  | Forward | 22.15 | 1.13 | 77.41 | 19.38 | 18.21 ± 0.47 |  |
| CsFAMA (Target) | Reverse | 22.82 | 1.18 | 81.54 | 21.96 | 21.11 ± 0.39 | 0.014 |
|  | Forward | 22.78 | 1.17 | 81.21 | 21.64 | 20.78 ± 0.42 |  |

^a)^The average device performance with standard deviation were obtained on the basis of 40 cells.

^b)^The hysteresis index (HI) is defined by [HI = 1– *J*_FS_(0.8*V*_OC_)/*J*_RS_(0.8*V*_OC_)], where *J*_RS_(0.8*V*_OC_) and *J*_FS_(0.8*V*_OC_) represent photocurrent density at 80% of *V*_OC_ for the RS and FS, respectively.

**Fig. S10. (**a) External quantum efficiency (EQE) spectra of the champion MAPbI_3_-based control (with PAT additives) and target (with Au@PAT additives) device. (b) Light absorbance of control and target MAPbI_3_ films. (c) Relative change ratios of EQE (ΔEQE) and absorption (Δabsorption) caused by the incorporation of plasmonic NPs in MAPbI_3_.

**Fig. S11.** (a) Current density–voltage (*J–V*) curves of the champion CsFA devices with PAT (Control) or with Au@NP additives (Target) under one Sun illumination. (b) EQE spectrum and *J*_SC_ integrated from the EQE spectrum of the control and target CsFA devices. (c) Light absorbance of CsFA perovskite films doped with PAT or with plasmonic NPs. (d) Relative change ratios of EQE (ΔEQE) and absorption (Δabsorption) caused by the incorporation of Au@PAT in CsFA.

**Fig. S12.** (a) Current density–voltage (*J–V*) curves of the champion CsFAMA devices with PAT additives (Control) and with Au@PAT additives (Target) under one Sun conditions. (b) EQE spectrum and *J*_SC_ integrated from the EQE spectrum of the champion control and target CsFAMA devices. (c) The EQE enhancement (ΔEQE) for CsFAMA devices.


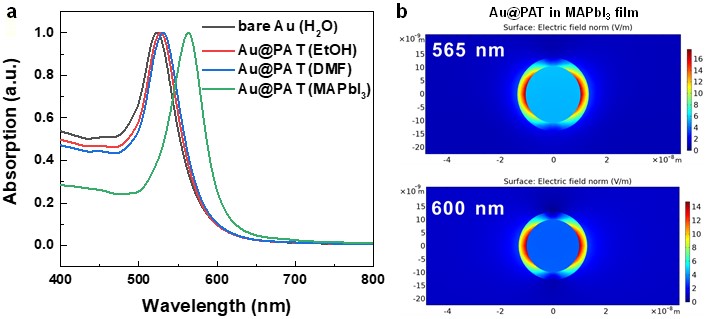


**Fig. S13.** (a) The simulated absorption of bare Au and Au@PAT NPs in both solution and solid states. (b) The electric field distributions at different wavelength for the MAPbI_3_ devices doped with Au@PAT NPs.

**Fig. S14.** (a) Steady-state photoluminescence (PL) spectra and (b) time-resolved PL spectroscopy of pristine, control (with PAT) and target (with Au@PAT) MAPbI_3_ films under excitation at 532 nm under low excitation ﬂuence (1 µJ cm^−2^). Monomolecular recombination rate constants (*k*_1_) are extracted from monoexponential fits to the PL decay at the time scale of hundred nanoseconds.

The PL decays of MAPbI_3_ perovskite films on the glass substrate show bi-exponential decays with fast (*τ*_1_) and slow components (*τ*_2_). We assign *τ*_1_ as a decay component closely related to non-radiative recombination by defects (such as trap at grain boundaries) and *τ*_2_ as a component of radiative recombination from bulk perovskite. The film modified with Au@PAT possesses much longer *τ*_1_, attributed to suppressed non-radiative recombination at grain boundaries. However, the similar *τ*_2_ value suggests a preserved film quality of bulk perovskite.

**Fig. S15.** Open-circuit voltage versus light intensity for the pristine, control and target MAPbI_3_ PSCs.

**Fig. S16.** XRD spectra of control and target MAPbI_3_ films exposed to ambient environment (30% relative humidity, 25 °C) for 48 h under visible light. Peaks labelled # correspond to PbI_2_.

**Fig. S17.** (a) Comparison of operational stability of encapsulated control and target (Cs_0.17_FA_0.83_)Pb(I_0.8_Br_0.2_)_3_ (CsFA) PSCs following ISOS-L-3 protocol (~65°C, 60% RH). After 400 h of continuous operation under AM1.5 illumination, the control CsFA device exhibited a reasonable decay by remaining 84% of its initial efficiency. In contrast, the target device demonstrated enhanced stability and retained over 95% of their initial PCE after the MPP test. (b) UV-visible absorption of control and target CsFA perovskite films before and after annealing in air at 85 ^o^C for 48 hours under visible light.

**Fig. S18.** (a) Top-view SEM images showing the evolution of film morphology of the pristine MAPbI_3_ film and the MAPbI_3_ films consisting of 0.4, 0.8, 1.2 and 2.0 mg ml^-1^ of Au@PAT NPs. The control MAPbI_3_ film with 0.4 mg ml^-1^ PAT is also shown. (b) The apparent grain size distribution for pristine, control and modified perovskite films measured from corresponding top-view SEM images.

Note: We speculate that the surface amino groups of PAT and Au@PAT interact with the Pb^2+^ and then act as the heterogeneous nucleation sites to facilitate the growth of perovskite crystals at a low concentration. However, excess amount of Au@PAT NPs (over 1.0 mg ml^-1^) would provide too many nucleation sites, resulting in significantly decreased grain size. This is consistent with the fact that further increasing the concentration of NPs to 2.0 mg ml^-1^ leads to a severe PCE drop.

**Fig. S19.** (a) XRD patterns of pristine and modified MAPbI_3_ films with various concentrations of Au@PAT (0.8 and 2.0 mg ml^-1^) and with PAT (control). (b) GIWAXS patterns for the perovskite thin films formed with and without the incorporation of 0.8 mg ml^-1^ of Au@PAT NPs. The characteristic reflections, (110) and (220), presented slightly increased intensity and no obvious shift in diffraction angle in the thin films deposited with the addition of 0.8 mg ml^-1^ NPs.

**Fig. S20.** (a) Dark *J-V* characteristics of electron-only pristine, control and target MAPbI_3_ devices for estimating the SCLC defect concentration. We attributed the slight decrease of trap densities to the PAT shells and the uncertainty in the measurement. (b) Surface recombination velocity (SRV) extracted from the TRPL lifetime data on MAPbI_3_ films, according to estimated method described previously^[9]^. (c) Ultraviolet photoemission spectroscopy (UPS) spectra of pristine, control and target MAPbI_3_ films. Left panels: photoemission cut-off for the perovskite films, from which the vacuum level of the film is extracted. The work functions of all the perovskite films are around 3.90 eV. Right panels: UPS spectra of the top of occupied states. VBM level for all the perovskite films are around 1.45 eV.

**Fig. S21.** TPV results of control and target MAPbI_3_ PSCs. (a) Normalized TPV spectra of the devices at a fixed background illumination of 1.05 V. (b) The charge carrier lifetime extracted from the TPV spectra as a function of bias light intensity.

**Fig. S22.** (a, b) Local normalized steady-state photoluminescence spectra of control and target perovskite films at bright (grain interior) and dark regions (grain boundary) identified in the circles of **Fig. 3a**. The dark region is both red shifted and slightly broader than the PL spectrum collected at the bright region. (c) PL mapping images of pristine MAPbI_3_ films measured under 532 nm photo-excitation. (d) Normalized PL line scans across GBs in an average of four random places of perovskite film without additives.

**Fig. S23.** Carrier density dynamics and global fit of control and target perovskite films, obtained from TA spectra. With increasing fluence, bimolecular and Auger recombination lead to increasingly rapid initial decay components. For pump fluence of 6 and 40 μJ cm^-2^, the initial carrier concentration (*n*_0_) is calculated to be around 2.7 × 10^17^ and 1.8 × 10^18^ cm^-3^, respectively.

Note: The initial photo-generated carrier concentration *n*_0_ is estimated using the following
equation:

$$n_{0}=\frac{A(\lambda)\times F}{E_{\mathrm{ph}}(\lambda)\times W} (S4)$$

where *F* is the total incident fluence at excitation wavelength *λ* = 532 nm, photon energy *E*_ph_(λ)
≈ 3.74 × 10^-13^ μJ, sample thickness *W* ≈ 300 nm, *A*(λ) is the overall absorbance of the thin film.
When the absorption thickness is much smaller than *W*, the absorbance can be analytically
estimated by

$$A\left( \lambda\right)=1-R-T=\left( 1-e^{-\alpha\left( \lambda\right)W} \right)\cdot\left( 1-R \right) (S5)$$

where *R* and *T* are the reflectance and transmittance respectively, *α*(*λ*) is the absorption
coefficient of material.^[10]^ For the four lead-halide perovskite samples discussed here, the overall
absorbance is about *Α* (532 nm) ≈ 0.5 at 532 nm according to previous reports, where most
of the loss in absorption comes from surface reflections. Therefore, *n*_0_ is estimated as 1.8 ×
10^18^ cm^-3^ when *F* = 40 μJ cm^-2^.

**Fig. S24.** Normalized TA spectra of control and target MAPbI_3_ films, under 532 nm excitation with pump fluence of 6 µJ cm^-2^ (a-b) 15 µJ cm^-2^ (c-d), and 40 µJ cm^-2^ (e-f). Spectral broadening at early times (before 1 ps) indicates hot-carrier distributions. The arrows indicate the decay of negative TA feature below the bandgap.

Note: By definition, hot carriers have excess kinetic energies at least more than *k*_B_*T* (*E* – *E*_f_ >> *k*_B_T) above the conduction and below the valence bands for electrons and holes, respectively. Such hot carriers have higher carrier temperature, *T*_c_, than the crystal lattice, which dissipates in a very short period of time (hundreds of fs) through a phonon emission process. Since the hot carrier distribution follows the Boltzmann distribution, the *T*_c_ can be calculated by fitting the high energy tail of the TA spectra with a simple Maxwell–Boltzmann function.^[11]^

$$\frac{\Delta T}{T}\left[ E \right]\propto\frac{1}{1+exp\frac{E-E_{f}}{k_{B}T}}\approx exp\frac{E_{f}-E}{k_{B}T} (S6)$$

where *k*_B_ is the Boltzmann’s constant and *E*_f_ is the quasi-Fermi energy. In this measurement, the TA signal is proportional to the change in absorption coefficient (Δ*T*/*T* ∝ ΔA) and the thermalization to quasi-equilibrium carrier distributions occurs on a timescale of ≤ 100 fs.

**Fig. S25.** Calculated photoexcited carrier temperatures against time delay as a function of pump fluence (carrier densities vary from 2.7 × 10^17^ to 1.8 × 10^18^ cm^-3^). The carrier temperature *T*_c_ is obtained by fitting the high-energy tail of the bleaching spectrum above the band edge. In the fittings, the selected tails are from 1/3 of the maximum bleaching with a 0.25 eV length, between 1.75 eV and 2.0 eV.

Note: The signal at 1.58 eV is proportional to the occupation of sub-bandgap states created by bandgap renormalization, which is given by the product of Fermi distribution *f*(*E*, *T*_c_) and the unchanged density of state near the band edge. The change of the Fermi function with carrier temperature, ∂*f*/∂*T*_c_, can be approximated for large values of *T*c (which are present at early times after excitation) as^[12]^

$$\frac{\partial f(E, T_{c})}{\partial T_{c}}\propto\frac{\left( E-E_{f} \right)e^{\frac{E-E_{f}}{kT_{c}}}}{kT_{c}^{2}\cdot\left( 1+e^{\frac{E-E_{f}}{kT_{c}}} \right)^{2}}\propto-\frac{1}{T_{c}^{2}} (S7)$$

This relation can be evaluated from the measured values for ∂*f*/∂*T*_c_, that is, the derivative of the cooling curve (Fig. S24) and ∂*f*/∂*T*_c_, which is proportional to the derivative of the 1.58 eV TA kinetics (Table S3). The negative TA feature below the bandgap, around 1.58 eV, has been attributed to various effects,^[13]^ yet a clear understanding is crucial to differentiate the excitonic or free charge-carrier nature of these materials.

**Table S3.** Fluence dependence of the carrier cooling time *τ*_Tc_ and transient absorption decay time *τ*_ΔT/T_ at 1.58 eV for the control and target MAPbI_3_ films.

| *N* (10^17^ cm^-3^) | 2.7 | 4.1 | 6.8 | 12.2 | 18.0 |
| --- | --- | --- | --- | --- | --- |
| *τ*_Tc_ (control) | 260 fs | 310 fs | 350 fs | 410 fs | 480 fs |
| *τ*_Tc_ (target) | 330 fs | 390 fs | 470 fs | 560 fs | 660 fs |
| *τ*_ΔT/T_ (control) | 320 fs | 340 fs | 360 fs | 390 fs | 410 fs |
| *τ*_ΔT/T_ (target) | 430 fs | 450 fs | 490 fs | 510 fs | 540 fs |

Note: As shown in Supplementary Fig. S24, the high energy tails of the photobleaching (PB) peak originate from the rapid distribution of initial non-equilibrium carriers into a Fermi-Dirac distribution via elastic scattering^[11]^. The relaxation of the hot carriers largely depends on the excitation conditions and the ﬂuence-dependent carrier temperatures (*T*_c_) as a function of time delay *t* are summarized in Supplementary Fig. S25 and Supplementary Table S3. Notably, our target perovskite film exhibited slightly longer hot-carrier cooling lifetimes than those of the control perovskite sample under similar *n*_0_. In addition, we noticed a sub-bandgap transient absorption signal at 1.58 eV, which is explained by the interplay of bandgap renormalization and hot-carrier distributions^[12]^. For each given carrier density, the kinetics of the sub-bandgap feature also shows slower decay with incorporation of Au@PAT in the MAPbI_3_ film.

Hot carriers will mainly lose their excess energy through inelastic electron–phonon coupling interactions. There are generally two coupling mechanisms that drive electron–phonon scattering in inorganic semiconductors: polar Fröhlich interactions of electron–LO phonon scattering and electron-acoustic phonon scattering, both of which are representative of the material’s intrinsic properties. Hot-carrier harvesting is compromised by competitive relaxation pathways (for example, intraband Auger process and defects) that overwhelm their phonon bottlenecks. Thus, the slower carrier relaxation observed in the Au@PAT-doped perovskite film convinces us that a large number of defects are well passivated in this sample, which suppresses relaxation pathways for the hot carriers.

**Scheme S2.** (a) Scheme of the PIRET. The plasmon decays with formation of an electron in the conduction band (CB) and a hole in the valence band (VB) of the MAPbI_3_. (b) Scheme of the PHET. A photoexcited plasmon decays into an electron–hole pair in the Au NP, followed by the transfer of the hot electron into the CB of MAPbI_3_ as indicated by the arrow.


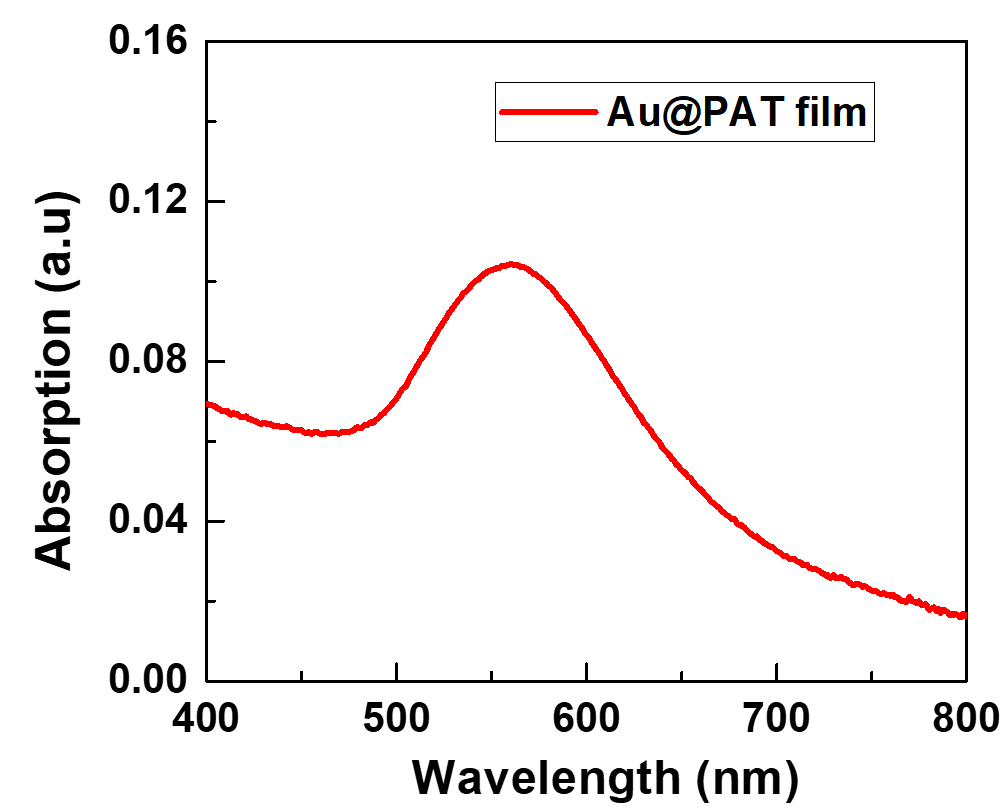


**Fig. S26.** Extinction spectra for self-assembly Au@PAT film on glass.

***Self-Assembly of Au@PAT film***: Large-scale assembly of the modified Au NPs on quartz glass substrates was done using a modified protocol^[14]^. Plain quartz glass slides are sequentially cleaned in ultrasonic baths of detergent solution, DI water, acetone and isopropanol for 15 min each. Next, quartz glass microscope slides were pretreated with Piranha solution (H_2_O_2_: H_2_SO_4_ 1:3, v/v) for half an hour and then immersed in a 2-propanol solution of 1% 3-aminopropyltrimethoxysilane (APTMS) in the presence of 0.1% acetic acid for 5 h. The modified substrates were rinsed with isopropanol three times prior to use. Finally, they are dipped into the as-prepared Au@PAT NPs solution for 24 h. The substrates coated with the NPs are rinsed with DMF to remove the unbound particles, dried under nitrogen and are then ready for use.

**Fig. S27.** Charge dynamics probed at the 580 nm in the samples of MAPbI_3_ film (0.2 M) deposited on bare glass substrate (*λ*_ex_ = 532 nm). The broad PIA band is assigned to photoinduced refractive index changes. The relaxation dynamics of PIA band in bare MAPbI_3_ film is significantly different with that of Au@PAT/MAPbI_3_ sample.

**Table S4.** The corresponding formation time constant (*τ*_et_) and hot electron transfer kinetics shown in Fig. 5e. The kinetics are fitted by a multiple-exponential function, ∆A(t) = a_1_exp(-*t*/*τ*_1_) +a_2_exp(-*t*/*τ*_2_)– exp(-*t*/*τ*_et_), where a_1_ and a_2_ are amplitudes; *τ*_1_ and *τ*_2_ are decay time constants and *τ*_et_ is formation time constant.^[15]^ The process of *τ*_1_ and *τ*_2_ account for electron-to-phonon and hot phonon relaxation processes, respectively.

|  | *τ*_et_/ps | *τ*_1_/ps (a_1_) | *τ*_2_/ps (a_2_) | *τ*_avg_/ps |
| --- | --- | --- | --- | --- |
| in Au@PAT/MAPbI_3_  bilayer (580 nm) | 0.21 | 22.5 (61.5%) | 516 (38.5%) | 212 |
| in Au@PAT (580 nm) | 0.12 | 1.89 (100%) | - | 1.89 |

**Fig. S28.** (a) Pseudo-colour TA spectra plot of Au@PAT/MAPbI_3_ bilayer film excited at 365 nm with a fluence of 15 μJ cm^-2^. (b) Charge dynamics probed at the 580 nm in the bilayer sample.

**Fig. S29.** PLE spectra recorded from bare MAPbI_3_ film and Au@PAT/MAPbI_3_ bilayer film. The spectra are normalized to the PL intensity under 750 nm excitation. The spectral trend of PLE curve exhibited two clear maxima at 510 and 750 nm, which coincide with the transition between a dual valence band and a conduction band.

**References**

[1] Q. Wang, Y. Shao, Q. Dong, Z. Xiao, Y. Yuan and J. Huang, Large fill-factor bilayer iodine perovskite solar cells fabricated by a low-temperature solution-process. *Energy Environ. Sci.*, 2014, ***7***, 2359-2365.

[2] S. Khelifi, K. Decock, J. Lauwaert, H. Vrielinck, D. Spoltore, F. Piersimoni, J. Manca, A. Belghachi and M. Burgelman, Investigation of defects by admittance spectroscopy measurements in poly (3-hexylthiophene):(6,6)-phenyl C_61_-butyric acid methyl ester organic solar cells degraded under air exposure. *J. Appl. Phys.,* 2011, **110**, 094509.

[3] T. Walter, R. Herberholz, C. Müller and H. W. Schock, Determination of defect distributions from admittance measurements and application to Cu(In,Ga)Se_2_ based heterojunctions. *J. Appl. Phys.*, 1996, **80**, 4411–4420.

[4] G. Kresse and J. Furthmüller, Efficient iterative schemes for ab initio total-energy calculations using a plane-wave basis set. *Phys. Rev. B*, 1996, **54**, 11169.

[5] G. Kresse and D. Joubert, From ultrasoft pseudopotentials to the projector augmented-wave method. *Phys. Rev. B*, 1999, **59**, 1758.

[6] J. P. Perdew, K. Burke and M. Ernzerhof, Generalized gradient approximation made simple. *Phys. Rev. Lett.*, 1996, **77**, 3865.

[7] W. Tang, E. Sanville and G. Henkelman, A grid-based Bader analysis algorithm without lattice bias. *J. Phys.: Condens. Matter*, 2009, **21**, 084204.

[8] Z. Chen, M. Liu, Z. Li, T. Shi, Y. Yang, H. L. Yip and Y. Cao, Stable Sn/Pb-based perovskite solar cells with a coherent 2D/3D interface. *iScience*, 2018, **9**, 337-346.

[9] J. Wang, W. Fu, S. Jariwala, I. Sinha, A. K. Y. Jen, D. S. Ginger, Reducing Surface Recombination Velocities at the Electrical Contacts Will Improve Perovskite Photovoltaics. *ACS Energy Lett.* **2018,** *4*, 222-227.

[10] J. Yang, X. Wen, H. Xia, R. Sheng, Q. Ma, J. Kim, P. Tapping, T. Harada, T. W. Kee, F. Huang, Y. B. Cheng, M. Green, A. Ho-Baillie, S. Huang, S. Shrestha, R. Patterson and G. Conibeer, Acoustic-optical phonon up-conversion and hot-phonon bottleneck in lead-halide perovskites. *Nat. Commun.*, 2017, **8**, 14120.

[11] Y. Yang, D. P. Ostrowski, R. M. France, K. Zhu, J. van de Lagemaat, J. M. Luther and M. C. Beard, Observation of a hot-phonon bottleneck in lead-iodide perovskites. *Nat. Photonics*, 2015, **10**, 53-59.

[12] M. B. Price, J. Butkus, T. C. Jellicoe, A. Sadhanala, A. Briane, J. E. Halpert, K. Broch, J. M. Hodgkiss, R. H. Friend and F. Deschler, Hot-carrier cooling and photoinduced refractive index changes in organic-inorganic lead halide perovskites. *Nat. Commun.*, 2015, **6**, 8420.

[13] K. Chen, A. J. Barker, F. L. C. Morgan, J. E. Halpert and J. M. Hodgkiss, Effect of carrier thermalization dynamics on light emission and amplification in organometal halide perovskites. *J. Phys. Chem. Lett.*, 2015, **6**, 153.

[14] C. Xue, Z. Li and C. A. Mirkin, Large-scale assembly of single-crystal silver nanoprism monolayers. *Small*, 2005, **1**, 513-516.

[15] K. Wu, W. E. Rodríguez-Córdoba, Y. Yang and T. Lian, Plasmon-induced hot electron transfer from the Au tip to CdS rod in CdS-Au nanoheterostructures. *Nano Lett.*, 2013, **13**, 5255-5263.
